# Histopathological Correlation of Breast Carcinoma with Breast Imaging-Reporting and Data System

**DOI:** 10.21315/mjms2022.29.4.7

**Published:** 2022-08-29

**Authors:** Suraya Aziz, Muhammad Afiq Mohamad, Reena Rahayu Md Zin

**Affiliations:** 1 Department of Pathology, Faculty of Medicine, Universiti Kebangsaan Malaysia Medical Centre, Kuala Lumpur, Malaysia; 2 Department of Radiology, Faculty of Medicine, Universiti Kebangsaan Malaysia Medical Centre, Kuala Lumpur, Malaysia

**Keywords:** BI-RADS, breast cancer, correlation

## Abstract

**Background:**

Breast cancer is one of the commonest malignancy cancer worldwide and the Breast Imaging-Reporting and Data System (BI-RADS) classification has been extensively utilised as an adjunct to histopathological examination for malignant breast diseases. This study aims to analyse the concordance between radiological and histopathological findings, demonstrate the high predictive value in the BI-RADS category and evaluate the impact of these findings on surgical intervention and treatment outcome.

**Methods:**

This is a single-centre retrospective study, analysing patients who underwent radiological examination with BI-RADS categories 3, 4 and 5 followed by histopathological examination confirming the diagnosis based on breast core biopsy or excision specimen over 3 years.

**Results:**

A total of 316 specimens from 310 patients were included in this study; 75 cases were categorised BI-RADS 3, 166 as BI-RADS 4 and 75 as BI-RADS 5. Of these, 66 (20.8%) patients in BI-RADS category 3, 82 (25.9%) in BI-RADS category 4 and 5 (1.6%) in BI-RADS category 5 were reported as benign on histopathological examination. Malignant cases were reported in nine (2.8%) cases in BI-RADS category 3, 84 (26.6%) in BI-RADS category 4 and 70 (22.2%) in BI-RADS category 5. The positive predictive value (PPV), negative predictive value (NPV), sensitivity and specificity were 63.9%, 88%, 94.48%, and 43.14%, respectively.

**Conclusion:**

There is a significant correlation between BI-RADS score and histopathological results of breast cancer. A higher BI-RADS score is associated with a higher possibility of malignancy (*P* < 0.001). Our institution’s performance is comparable to other previously published data.

## Introduction

Invasive breast carcinoma is composed of a diverse group of malignant epithelial neoplasms of the glandular origin of the breast. Its incidence rate has been rising over the past few decades in Malaysia, probably due to the increased detection rate contributed by effective screening. However, breast carcinoma remains a major cause of morbidity and cancer-related mortality among women.

In Malaysia, a total of 103,507 new cancer cases were diagnosed between 2007 and 2011; of them, 54.8% were reported in females. Breast cancer was ranked as the commonest cancer which accounts for 34% of all cancers reported in Malaysia (1). The report showed that Chinese, followed by Indian and Malay ethnic groups, recorded the highest incidence of breast cancer. The 5-year survival rate of breast cancer patients in Malaysia was 49% (median interval, 68.1 months) (2). However, the overall 5-year survival rate of Malaysian breast cancer patients was comparatively lower than in many other developed countries (3). Similar lower rates were reported in other Asian continents compared to the Western countries (4).

A breast lump is the most common presentation associated with benign or malignant breast lesions. The detection of breast lesions has shown significant improvement over recent years due to advanced imaging studies. Mammogram and ultrasound are the two non-invasive, affordable, widely available radiological interventions that aid in the diagnosis and play a key role in early detection, treatment and favourable prognosis, resulting in improved survival rates in breast cancer patients (5).

The Breast-Imaging and Reporting Data System (BI-RADS) has been implemented widely for reporting in mammography and more recently in breast ultrasound. The reporting is done by giving a BI-RADS category score, duly recommending further management. Details of the BI-RADS for mammogram and ultrasound are as follows: i) category 0 - incomplete assessment; ii) category 1 - negative; iii) category 2 - benign finding(s); iv) category 3 - probable benign findings; v) category 4 - suspicious abnormality; vi) category 5 - highly suggestive of malignancy and vii) category 6 - known biopsy-proven malignancy. BI-RADS category 3 has the lowest probability of malignancy (< 2%). BI-RADS category 4 is predictive of breast cancer at approximately 30% and BI-RADS category 5 has the highest likelihood of malignancy at more than 95%. The BI-RADS category 4 is divided into BI-RADS 4a, 4b and 4c subcategories to stratify the risk of malignancy. Based on the category given, the American College of Radiology (ACR) recommends a different approach to management (6). For BI-RADS categories 1 and 2, an annual mammogram screening programme is suggested. A short interval follow-up within 6 months is recommended for BI-RADS category 3 whereas for BI-RADS categories 4 and 5, tissue diagnosis is suggested.

Although the BI-RADS category has been implemented in the reporting of ultrasound and mammograms in healthcare institutions nationwide, studies are scarce on evaluating our performance achieved in Malaysia. In the current study, we evaluated the results of our institution’s radiological assessment and correlated them to the final diagnosis by histopathological examination. The study also determined the sensitivity, specificity and accuracy of the assessment. Overall, this study aims to demonstrate the high predictive value of the BI-RADS categories and evaluate their impact on surgical intervention and treatment outcomes.

## Methods

This single-centre retrospective study was conducted after obtaining prior approval from our Institutional Research Ethics Committee, Faculty of Medicine, Universiti Kebangsaan Malaysia (UKM). The patients who had undergone radiological imaging either ultrasound or mammography with a BI-RADS scoring system and histopathological examination confirming the diagnosis of breast carcinoma were enrolled. Data of patients who fulfilled the inclusion criteria were collected via Integrated Laboratory Management System (ILMS) software, Integrated Radiology Information System (IRIS) software and the Universiti Kebangsaan Malaysia Medical Centre (UKMMC) Caring Hospital Enterprise System (CHETS) from 2016 to 2018. Based on the prevalence of breast carcinoma in Malaysia, 224 cases were required for the study (using Kish L. 1965 formula for sample size calculation).

The patients selected were classified into BI-RADS categories 3–5. BI-RADS category 3 was considered most likely benign and BI-RADS categories 4–5 were taken as malignant. The demographic was reviewed including the patient’s age, ethnicity, presenting symptoms, menopausal status and histopathological diagnosis, as well as pathological reports. The tissue diagnosis was performed by either core breast biopsy, wide local excision or mastectomy.

Exclusion criteria included the following cases:

- Patients with no concomitant radiological and histopathological examination
- Having insufficient clinical data or medical records
- Those who scored BI-RADS categories 0–2 as well as BI-RADS category 6

Benign breast lesion constitutes a heterogeneous group of diseases arising from different histological origins which include epithelial, stromal or other mammary tissues. For this study, benign and borderline phyllodes tumours were categorised under benign lesions. According to AJCC Cancer Staging Manual 8th edition, ductal carcinoma in situ (DCIS) is considered a precursor lesion of breast carcinoma whereas lobular carcinoma in situ (LCIS) is now a benign entity.

The data were analysed using SPSS version 26 software. The quantitative data were compared and evaluated using descriptive statistics (mean, median and standard deviation) in addition to the chi-squared test as the test of significance. Standard computation of sensitivity, specificity, positive predictive value (PPV) and negative predictive value (NPV) was done, along with their confidence intervals at 95%.

## Results

A total of 316 specimens from 310 patients were collected during a 3-year window period. Six patients underwent bilateral breast biopsies. The chief presenting complaint was a palpable breast lump. Non-lump breast symptoms such as breast pain and nipple discharge were less commonly encountered. The age of the study population ranged from 18 years old to 84 years old (mean = 53 years old). Next, 103 malignant cases (66%) were reported from the post-menopausal period. In terms of ethnicity, the Malay ethnic group recorded the highest incidence of malignant breast cancers (63.3%), followed by the Chinese and the Indians. Other Malaysian ethnic groups and foreigners who sought treatment in the UKMMC constituted 4.7% of the total cases (Table 1).

When categorised according to either benign or malignant cases, post-menopausal women were associated with significantly higher malignant cases (*P* < 0.001) with higher overall BI-RADS category 5. There were 120 core breast biopsies performed without subsequent excision. Ninety-two of the biopsies were reported as benign while the remaining 28 were malignant. All of the patients diagnosed with malignant breast disease were recommended for excision and a total of 196 patients underwent excision by either wide local excision or mastectomy. Of the 316 specimens, 75 cases were reported into BI-RADS category 3, 166 as BI-RADS category 4 and 75 as BI-RADS category 5. All patients underwent either core breast biopsy or excision biopsy. The lesions were examined by histopathological examination and were determined as benign breast tissue or lesions in 66 cases that received a BI-RADS category 3 score, 82 cases classified as BI-RADS category 4 and 5 cases in BI-RADS category 5. Malignant breast lesions were detected in 9 cases reported being BI-RADS category 3, 84 as BI-RADS category 4 and 70 as BI-RADS category 5, respectively. Table 2 summarises the frequency of BI-RADS.

The malignant cases in BI-RADS category 3 were five (6.7%) ductal carcinoma in situ, three invasive carcinomas of no special type (4.0%) and one solid papillary carcinoma with invasion (1.3%). In these cases, no or vague masses were detected in two cases, one displayed intracystic mural nodule, one had ill-defined heterogeneous mass and five cases were described as well-defined hypoechoic lesions. Lesions with masses described in the radiology report measured between 0.4 cm and 2.2 cm. Coarse calcifications with marginal projections were found in one case. No microcalcifications were observed. Five patients had both ultrasound and mammogram assessments performed. Some of the patients refused mammogram examination, stating that breast pain during procedure was the main concern.

Five benign cases were reported under BI-RADS category 5. They are one (1.3%) benign breast tissue, one (1.3%) fibrocystic change, one (1.3%) florid usual ductal hyperplasia, one (1.3%) borderline phyllodes tumour and one (1.3%) intraductal lesion favouring intraductal papilloma. Dual ultrasound and mammogram assessment were performed in one patient. The masses were partially circumscribed, irregular or ill-defined hypoechoic lesions, measuring between 0.4 cm and 3.4 cm. Only two cases demonstrated macro and microcalcifications changes each. Three patients underwent wide local excision and the final histologic features of each case in the excised tissue consisted of papilloma, borderline phyllodes and florid ductal hyperplasia. Only core biopsies were performed on the remaining two patients who were subsequently lost to follow-up.

The PPV for BI-RADS category 5 lesions for malignancy was 93.3% whereas the NPV of BI-RADS category 3 lesions for malignancy was 88.0%. BI-RADS category 5 is associated with a significantly higher frequency of malignant breast cases than BI-RADS category 4 (*P* < 0.001). Overall, the breast lesions evaluated by BI-RADS classification have a sensitivity of 94.48%, specificity of 43.14%, a positive predictive value of 63.9% and a NPV of 88%.

In terms of histopathological examination inconsistencies between initial core biopsy with a follow-up biopsy and excision specimen, five cases were in BI-RADS category 4 and four cases were in BI-RADS category 5. In BI-RADS category 4 cases, four biopsies were reported as non-representative of the underlying pathology and one biopsy was reported as a papillary lesion with usual ductal hyperplasia (UDH) with the suggestion of excision for definitive diagnosis. Two of the four discrepant cases were later reported as encapsulated papillary carcinoma with DCIS and invasive carcinoma of no special type. The third case had a repeat biopsy which was also described as benign breast tissue and she was continued for follow-up under breast endocrine clinic. This patient later developed invasive carcinoma of no special type. The fourth case was reported as a papillary lesion and UDH was confirmed to be mucinous carcinoma with DCIS. An intraoperative frozen section was requested in the final case due to high suspicion of malignancy, which confirmed the diagnosis of invasive carcinoma.

**Figure 1:**
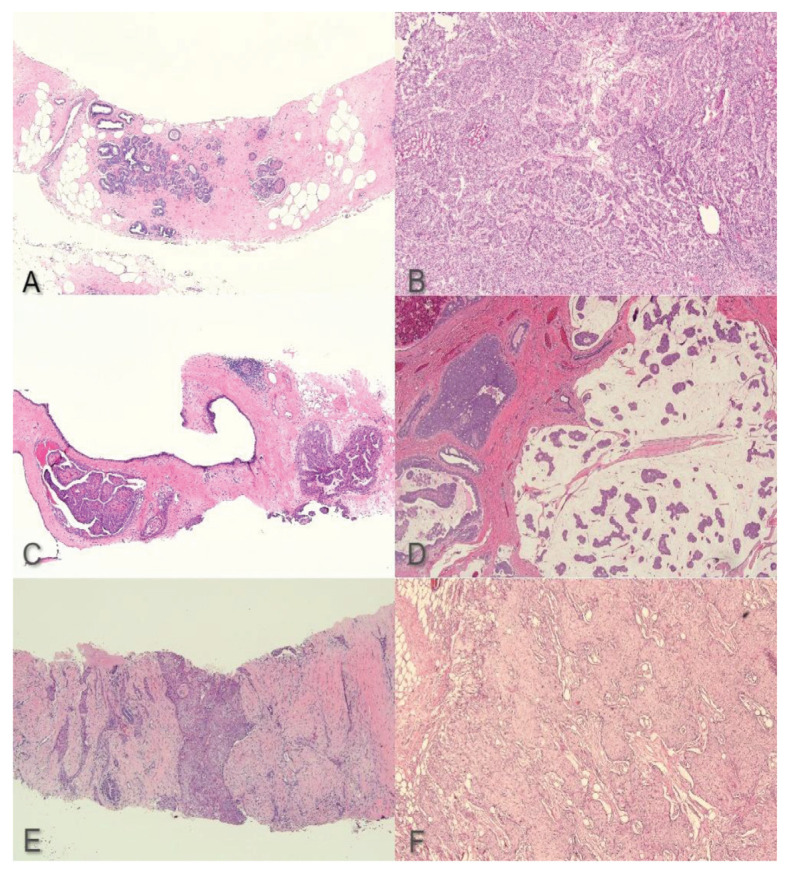
Images of cases with discrepancies between biopsy and excision specimen: (A) shows benign breast biopsy which was later confirmed to be invasive breast carcinoma of no special type on frozen section (B), (C) shows papillary lesion on breast biopsy with a mucinous breast carcinoma component found on excision specimen (D), (E) shows a breast biopsy which was initially reported as fibrous epithelial lesion with squamous metaplasia but later revised as metaplastic carcinoma (F). (haematoxylin and eosin, 1.25 and 4× magnification)

Four cases that showed the BI-RADS category 5 were initially thought to have discrepancies as three of these cases were reported as non-representative and one as a fibrous epithelial lesion with squamous metaplasia. The three non-representative cases mainly showed benign breast tissue on the initial biopsy. Due to high clinical suspicion, an intraoperative frozen section consultation was done for the first case which showed invasive carcinoma, thereby confirming wide local excision. The second and third cases underwent a repeat biopsy which showed invasive carcinoma. Due to malignant clinical features seen in the final discrepant case, a revised interpretation of the biopsy showed features of metaplastic carcinoma which was further confirmed upon receiving the mastectomy specimen (Figure 1).

The commonest malignant pathology diagnosed was invasive ductal carcinoma of no special type which accounts for 71.5% of the total malignant cases. This is followed by ductal carcinoma in situ at 7.9%, out of which two of these cases are associated with microinvasion. Invasive lobular carcinoma is the third-highest reported malignant breast lesion (6.1%), solid papillary carcinoma (2.4%), malignant phyllodes tumour (1.8%), metaplastic and mucinous carcinoma (1.2% each).

Benign breast tissue or fibrotic breast tissue was the most common diagnosis given for benign lesions (29.1%). The second most frequently encountered breast lesions were benign fibroepithelial lesions which include fibroadenoma (21.8%). Other lesions in this category are intraductal papillomas, adenomas, benign/borderline phyllodes tumour as well as a constellation of non-proliferative and proliferative changes such as epithelial hyperplasia, adenosis and cysts collectively termed as fibrocystic changes.

## Discussion

BI-RADS was first introduced and adopted by the ACR. BI-RADS reporting comprises a few key elements for standard reporting. First, the indication or main purpose of the study is either for screening, diagnostic or follow-up. This is followed by the overall breast composition. All reporting contains a statement regarding breast density based on the proportion of glandular and fatty tissues. The main body of the report mentions any abnormalities during the study. This includes a mass or a lesion, its shape, margin, density, calcifications and architectural distortion.

Prior to the BI-RADS scoring system, variability in terminology during radiological reporting has created confusion among clinicians. These have often led to misinterpretation and inconsistencies for further evaluation which may contribute to the prognosis and overall survival rate (7). BI-RADS scoring system aimed to standardise radiology reports when analysing breast imaging mainly for separating benign lesions from potentially malignant ones and make recommendations for further management.

According to BI-RADS^®^ (6), the malignancy rates range from 2% for BI-RADS category 3 lesions up to 95% for BI-RADS category 5. Few studies also showed that the PPV for BI-RADS category 5 can reach up to 100%, which is similar to our study. The overall sensitivity, specificity, positive predictive value and NPV were comparable to previous studies (8–11). Liberman et al. (8) in their study on 492 patients reported that the PPV for malignant lesions values in BI-RADS category 5 ranged from 81% to 97%. BI-RADS category 4 revealed a lower PPV, between 23% and 24%. Chotiyano et al. (12) documented that the PPV in 424 women for BI-RADS category 5 was 85%. This was in accordance with PPV suggested by American Cancer Research which was 95% and other studies that suggested a PPV value between 80% and 97%. Siegmann et al. (13) correlated the BI-RADS category and tissue breast biopsy in suspected malignant cases. Tissue core biopsies were performed on 132 patients with detected mammogram lesions. The malignancy rates increased from 6.3% in category 3 to 16.7% in BI-RADS category 4 and up to 85% in BI-RADS category 5. Hoti et al. (14) also supported the significant correlation between BI-RADS classification and histopathological results. An exception was made for BI-RADS category 3 in which the final diagnosis of one case was DCIS. Another study involving 97 patients recommended BI-RADS category 3 breast lesions should be followed up with tissue biopsy (14).

Both the American College of Radiology and the Society of Breast Imaging have recommended breast ultrasound as an adjunct to mammography. In our study, however, not all results were supported by both imaging. A prospective study done by Harini et al. (15) on 55 patients showed statistically significant *P*-values for mammogram and ultrasound BI-RADS reporting in discerning benign from malignant breast disease (15). Combining both modalities boast greater sensitivity and specificity for detecting breast lesions. Silva and Furtado (16) in a study of 110 cases reported that ultrasound features of breast lesions have a high predictive value, significantly influencing the recommended management and outcome. This study showed that all patients with radiological imaging with high suspicion of malignancy had a histopathological diagnosis of breast carcinoma. The majority of mammogram and ultrasound results in 105 patients reported as malignant, corresponds to the histopathological diagnosis of a malignant breast lesion, showing that mammogram was the preferred diagnostic tool for breast cancer screening in women aged > 40 years old (17).

Calcification is an important criterion in mammography as its morphology and distribution are correlated to the histology of the lesion. In some cases, breast calcifications may be the earliest sign of breast cancer development. Almost half of the nonpalpable breast cancer was detected through microcalcifications (18). Another study reported a higher detection rate for DCIS based on microcalcifications and showed that invasive breast carcinomas are usually associated with microcalcifications (19). The features that can be observed and yield the highest positive predictive values are spiculated margins, irregular outline, linear and segmental microcalcification (8). In the present study, coarse calcification was described in one malignant case in BI-RADS category 3, which is typically a benign feature with no microcalcification observed.

Discrepant cases between histopathological examinations reported as benign breast tissue or non-representative of the lesion were followed up by either a repeat biopsy or intraoperative frozen sections in our study. According to the EC Working Group on Breast Screening Pathology, a repeat biopsy or excision biopsy is indicated if there are inconsistencies between clinical and radiological examination with a biopsy composed entirely of normal breast tissue (20). Hence, precise targeting of the lesion and getting a good adequate tissue sample might reduce false-negative cases. Papillary lesions in a breast biopsy may be challenging to interpret, requiring further immunohistochemistry for confirmation. Even with the addition of ancillary studies, significant false positive and false negative rates are observed when making a diagnosis of papillary lesions (21). All of the cases in this study were followed up within a 6-month to 12-month period. Some of the patients diagnosed with malignancy received neoadjuvant chemotherapy prior to surgery. Other surgeons offered excision by either wide local excision or mastectomy to patients, followed by systemic therapy.

Age and menopausal status are also two key factors to consider in the development of breast cancer. In this study, 66% of post-menopausal women were associated with significantly more malignant cases compared to pre-menopausal and peri-menopausal periods. Malignancy rates generally increased with age as shown by 63.2% of those aged ≥ 50 years old (*P* < 0.001) were diagnosed with malignant breast disease. These results support previous findings in multiple studies in the Asian population whereby the mean age of diagnosis was 50.6 years old (22–23).

## Conclusion

Overall, our institute’s performance was comparable to other published data and results indicated a high predictive value of the BI-RADS classification in evaluating the likelihood of breast carcinoma in patients presenting with a breast lesion. This approach can be a very powerful predictor of malignancy in the hands of the experienced radiologist. Imaging with the BI-RADS classification should be used in conjunction with clinical examination and tissue biopsy to provide a comprehensive perspective in the early detection of the breast cancer.

## Figures and Tables

**Table 1:** The demographic of cases collected in 2016–2018

Demographic data table	*n* (%)
Age (years old)	
Min	18
Max	84
Mean	53.22
Race	
Malay	200 (63.3)
Chinese	75 (23.7)
Indian	20 (6.3)
Others	15 (4.7)
Menopausal status	
Pre-menopausal	118 (38.1)
Peri-menopausal	36 (11.6)
Post-menopausal	156 (50.3)
Histopathological diagnosis	
Malignant	163 (51.6)
Benign	153 (48.4)
BI-RADS scoring	
Category 3	75 (23.7)
Category 4	166 (52.5)
Category 5	75 (23.7)
Core biopsy	
Category 3	45 (60)
Category 4	60 (36.1)
Category 5	15 (20)
Excision	
Category 3	30 (40)
Category 4	106 (63.9)
Category 5	60 (80)

**Table 2:** The frequency of malignant and benign cases, corresponding to age group, menopausal status and BI-RADS scoring

	Holoprosencephaly diagnosis (%)		*P*-value^a^

	Malignant	Benign	
Age group			
Less than 50 years old	43 (34.1)	83 (65.9)	
50 years old and above	120 (63.2)	70 (36.8)	< 0.001

Total	163	153	< 0.001

Menopausal status			
Pre-menopausal	37 (31.1)	82 (68.9)	< 0.001
Peri-menopausal	21 (56.8)	16 (43.2)	< 0.001
Post-menopausal	105 (65.6)	55 (34.4)	< 0.001

Total	163	153	

Mammographic and ultrasound BI-RADS scoring			
Category 3	9 (12)	66 (88)	< 0.001
Category 4	84 (50.6)	82 (49.4)	< 0.001
Category 5	70 (93.3)	5 (6.7)	< 0.001

Total	120	196	

Note:

a *P*-value < 0.001 is significant
